# Dental Medicine

**Published:** 1891-12

**Authors:** 


### Bibliographical.

DENTAL MEDICINE: A Manual of Dental Materia Medica and Therapeutics, by F. J. S. Gorgas, A. M., M. D., D. D. S. Fourth edition. Revised and enlarged. Philadelphia: P. Blakiston, Son & Co., 1012 Walnut street. 1891.

This new edition of the work of our industrious confrere in Baltimore, proves that its author is determined to make it an indispensable text-book for the student, and a ready reference for the busy practitioner. Much new matter has been added, bringing the work up to the times. The diagnosis of the affections of the mouth, the remedial agents, the properties, actions, uses and modes of application of the substances classed as dental materia medica, are carefully presented; while new matter has been added on the use of antiseptics.

In the examination of students, whose education has been limited to the curriculum of the dental colleges, there is perhaps no subject in which more confusion, and frequently ignorance, is displayed, than in the branch of dental materia medica and therapeutics. Dr. Gorgas's work covers the field as fully as modern dental education demands; and no matter how much a man may know, or imagines he knows, on this particular subject, the volume will bear earnest study.—*Dominion Dental Journal.*

---

When, early in 1884, we were called upon editorially to notice the first edition of Professor Gorgas' work on Dental Medicine we said: "It is an encouraging sign when repeated text-books upon materia medica and therapeutics multiply, for it indicates that dentistry has outgrown the day when it was considered but a mechanic art." But how could we have anticipated that in seven short years the fourth edition of such a work would be demanded, every copy of previous editions having been exhausted? Yet here it is, enlarged so as to include the latest discoveries in dental therapeutics. The book has become, not only a recognized text-book in every dental school in the world, but a necessary feature in the library of every practitioner who makes any pretense to complete practice.

The time has long passed when it was necessary to recommend this book to dental students. Its place in our literature is secure, and the present duty of the critic is to point out any possible deficiencies, and to assist in making it what it aims to be—the standard recognized authority in its special field.

It seems time that we, as dentists, forsook the old beaten paths of medicine and struck out for ourselves. Why, in our special field, should we accept the pathological views of the long ago? Surely there has been sufficient advance all along the line for dentists to begin to think for themselves, and to reject that which their special studies should teach them is false. We take exception, for instance, to the following, found on page 41 of the work under notice:

"The irritation of teething is indicated by a hot, swollen and tender condition of the gums, fretfulness, irritable temper, refusal of nourishment, fever and thirst, and if not relieved, diarrhœa with offensive motions, sometimes a troublesome cough, convulsions and other serious results."

To our apprehension, here is a decided lack of clearness in symptomatology. The indications of some general disturbance, of stomatitis, and of digestive disorders, are all mingled with derangements of a possible dental origin, so that a clear diagnosis from this description would be extremely difficult. It has been the habit of physicians to attribute to teething the diarrhœa and dysenteries, the fevers and fretfulness, that are the results of injudicious feeding. The advancement of the teeth has long been a convenient makeshift for the incompetent or thoughtless practitioner, who, because diarrhœas occur about the time of the eruption of the teeth, ascribes them to that source, heedless of the fact that it is also the period at which the diet of the child is frequently changed. With about as much show of reason could the teething be attributed to the diarrhœa.

It matters little that the gastric disturbances may occur when no tooth is anywhere near its normal period for eruption.

The infant of six months may be fed on corned-beef and cabbage, and if a diarrhœa occurs it is attributed to the fact that its teeth may be coming, when there is not the first sign of their advancement.

The disturbances incident to teething are necessarily of a nervous character mainly, and the paragraphs succeeding the one which we have quoted, if carefully scanned, would seem to recognize this. We would have a book of the great importance of the one under notice clearly point out these facts, and not follow the antiquated teachings of the past.

We could instance what we believe to be confusion in describing other conditions, notably in the chapter on Inflammation, and in the Effects of Micro organisms, but perhaps we have said enough to indicate our meaning. Each edition of the work has been a distinct advance upon the previous ones, and when, a year or two hence, a fifth is called for, we shall expect to applaud great progress in this particular.

In the meantime, if the reader of this notice does not possess the book he must get it, or acknowledge that he is not fully equipped for his daily duties.—*Dental Advertiser.*

---

